# Population Decoding in Rat Barrel Cortex: Optimizing the Linear Readout of Correlated Population Responses

**DOI:** 10.1371/journal.pcbi.1003415

**Published:** 2014-01-02

**Authors:** Mehdi Adibi, James S. McDonald, Colin W. G. Clifford, Ehsan Arabzadeh

**Affiliations:** 1 School of Psychology, University of New South Wales, Sydney, New South Wales, Australia; 2 Eccles Institute of Neuroscience, John Curtin School of Medical Research, The Australian National University, Canberra, Australian Capital Territory, Australia; 3 School of Psychology & Australian Centre of Excellence in Vision Science, University of Sydney, Sydney, New South Wales, Australia; Indiana University, United States of America

## Abstract

Sensory information is encoded in the response of neuronal populations. How might this information be decoded by downstream neurons? Here we analyzed the responses of simultaneously recorded barrel cortex neurons to sinusoidal vibrations of varying amplitudes preceded by three adapting stimuli of 0, 6 and 12 µm in amplitude. Using the framework of signal detection theory, we quantified the performance of a linear decoder which sums the responses of neurons after applying an optimum set of weights. Optimum weights were found by the analytical solution that maximized the average signal-to-noise ratio based on Fisher linear discriminant analysis. This provided a biologically plausible decoder that took into account the neuronal variability, covariability, and signal correlations. The optimal decoder achieved consistent improvement in discrimination performance over simple pooling. Decorrelating neuronal responses by trial shuffling revealed that, unlike pooling, the performance of the optimal decoder was minimally affected by noise correlation. In the non-adapted state, noise correlation enhanced the performance of the optimal decoder for some populations. Under adaptation, however, noise correlation always degraded the performance of the optimal decoder. Nonetheless, sensory adaptation improved the performance of the optimal decoder mainly by increasing signal correlation more than noise correlation. Adaptation induced little systematic change in the relative direction of signal and noise. Thus, a decoder which was optimized under the non-adapted state generalized well across states of adaptation.

## Author Summary

In the natural environment, animals are constantly exposed to sensory stimulation. A key question in systems neuroscience is how attributes of a sensory stimulus can be “read out” from the activity of a population of brain cells. We chose to investigate this question in the whisker-mediated touch system of rats because of its well-established anatomy and exquisite functionality. The whisker system is one of the major channels through which rodents acquire sensory information about their surrounding environment. The response properties of brain cells dynamically adjust to the prevailing diet of sensory stimulation, a process termed sensory adaptation. Here, we applied a biologically plausible scheme whereby different brain cells contribute to sensory readout with different weights. We established the set of weights that provide the optimal readout under different states of adaptation. The results yield an upper bound for the efficiency of coding sensory information. We found that the ability to decode sensory information improves with adaptation. However, a readout mechanism that does not adjust to the state of adaptation can still perform remarkably well.

## Introduction

**Figure 1:**
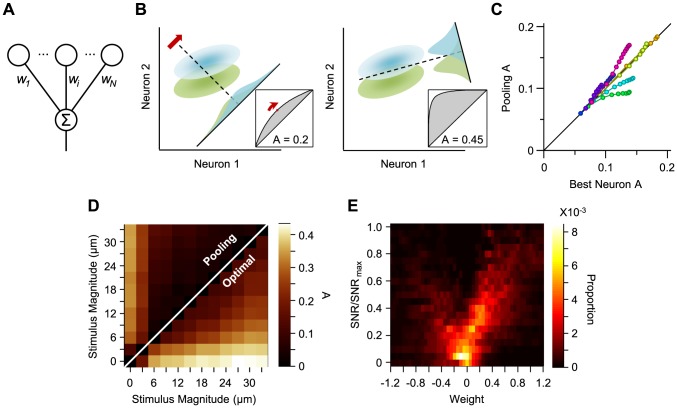
Population decoding. **A.** Schematic representation of linear combination of neuronal activity by the downstream decoder. Coefficients 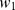, 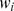 and 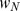 represent the synaptic weights between the neurons (top row circles) and the decoder (bottom). **B.** Schematic representation of pooling (left panel) and optimal decoding (right panel). The green and blue ovals represent the joint distribution of the neurons' responses to two sensory stimuli. The solid black line represents the weight vector. The pooling method (left panel) is equivalent to a weight vector along the identity line. The bell-shaped areas on the weight vector represent the projection of the neuronal response distribution for each stimulus. Dashed lines correspond to the best criterion to discriminate the two stimuli. The insets show the hit rate versus false alarm rate (ROC) for every possible criterion, shading indicates area *A*. **C.** Average value of *A* for the pooled neuronal responses plotted against the average value of *A* for the best neuron. Various population sizes within a session are plotted with the same color and connected with a line. For each population size, the value of *A* is averaged across all possible selections of that size. **D.** The average value of *A*, for each stimulus pair, under pooling (upper triangle) and optimal decoding (lower triangle), across all populations of 8 single neurons. **E.** Histogram of the optimal weights as a function of the signal-to-noise ratio of the same neuronal populations as in **D.** The weights and SNR values are normalized to the best neuron in each population.

A goal of systems neuroscience is to achieve a quantitative understanding of how cortical neurons report sensory events in their population activity. The interlaced synaptic architecture of neuronal networks provides anatomical evidence for population decoding by downstream neuronal structures. Such a synaptic organization allows an integration model in which the activity of neurons in the relevant population is summed with different weights. Under this model, discrimination of different stimuli can be formalized in terms of a linear classification of the neuronal responses. Here, we use a biologically plausible method of decoding: the model downstream neuron (the decoder) assigns a weight to each neuron before integrating the population activity (Figure 1A). The weight coefficient represents the synaptic strength between the input neuron and the decoder. This allows us to define an optimal linear decoder and establish its dependence on the adapted state of the network and its tolerance to correlated trial-to-trial covariability across neurons (noise correlation [1]–[4]).

In a recent study, we found that sensory adaptation improves coding efficiency of single neurons and the summed activity across neurons [5]. The present paper reanalyzes the same dataset with a focus on *decoding*. Investigating the behavior of the system under different adaptation states allows us to compare the performance of a non-adaptive decoder, which is optimal only under the non-adapted state, and an adaptive decoder, which adjusts to network dynamics and is thus optimal for any state of adaptation. In addition, by decoding simultaneously recorded single neurons, we quantify the influence of signal and noise correlations on the information available to downstream neurons.

## Methods

### Ethics statement

All components of the experiment were conducted in accordance with international guidelines and were approved by the Animal Care and Ethics Committee at the University of New South Wales (ACEC 08/77B and 10/47B).

### Surgery, electrophysiology and stimulation

For the present study we reanalyzed the recorded neuronal data in [5]. A brief description of the recording method follows. Six adult male Wistar rats were used for acute recordings. Anesthesia was induced by intra-peritoneal administration of Urethane (1.5 gr/kg body weight). Neuronal activity was acquired using a 32-channel 4-shank multi-electrode probe (NeuroNexus Technologies, Ann Arbor, MI) from the barrel cortex. The stimulus train was composed of a 250 ms adaptation stimulus of 80 Hz sinusoidal vibration followed by a half-cycle (6.25 ms) pause and a single-cycle sinusoidal test stimulus (frequency of 80 Hz, 12.5 ms). We used 10 blocks at each of 3 adaptation amplitudes (0, 6 and 12 µm). Each block contained twelve test stimuli (amplitudes of 0 to 33 µm with equal increment steps of 3 µm) presented in a random order. Throughout a recording session each test stimulus was repeated 100 times under every adaptation state. Neuronal response to different stimulus amplitudes was characterized by counting the number of spikes generated in each trial over a 50 ms window post stimulus onset. Previous recordings from barrel cortex have revealed that most of the information about vibration stimuli is transmitted within this time window [6], [7]. In 6 male rats, a total of 73 single units and 86 multi-unit clusters were recorded across a total of 16 sessions (see Table 1 in [5]). Each session contained a distinct set of simultaneously recorded neurons that were isolated using an online amplitude threshold and an offline template-matching procedure.

### Receiver operating characteristic (ROC) analysis

To explore population decoding, we quantify the discriminability obtained from (i) the pooled activity of simultaneously recorded neurons (i.e. all spike counts summed together), and (ii) the population activity of neurons when they are integrated after applying an optimum set of weights. For a population of *N* neurons, the spike counts are represented as a data point in an *N*-dimensional space where every dimension corresponds to a neuron in the population. Each data point is then projected onto the given weight vector. Pooling gives equal weights to all neurons such that the weight vector lies along the identity line (Figure 1B, left panel). An optimum linear decoder assigns different weights to neurons based on an algorithm (detailed below) to provide maximal separation between the response distributions (Figure 1B, right panel). Once the weight vector is determined, population response histograms are calculated from the projection of data points onto the weight vector. The overlap between the two histograms is quantified by applying an ROC analysis considering all possible values of the decision criterion, ranging from the minimum to the maximum observed projection values (see left panel in Figure 1B). Each criterion yields a hit rate and false-alarm rate; plotting the hit rates versus the false alarm rates leads to an ROC curve (insets in Figure 1B). Here we use the area (denoted by *A*) between the ROC and the identity (non-discriminant) line. The area *A* is calculated by approximating the missing parts of the ROC curve between two consecutive criteria by a trapezoid. The value of *A* falls within the range of 0 to 0.5; *A* = 0 indicates that the hit rate is equal to the false alarm rate, reflecting complete overlap between two histograms, thus no discriminability. *A* = 0.5, on the other hand, indicates no overlap between the two histograms and thus perfect discriminability. The value of *A* takes into account the trial-by-trial variability in response and characterizes discrimination performance supported by the neuronal population. For the whole stimulus set, the overall discriminability was defined as the average value of *A* across all possible pairwise comparisons of stimuli (n = 66).

### Fisher linear discriminant analyses

In order to identify the optimum weight vector for population decoding, we applied Fisher linear discriminant analysis [8]–[10] on the neuronal spike counts. For a population of *N* neurons, let the *N*×100 matrix 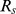 denote the neuronal responses to stimulus *s* across 100 trials, and the *N×N* matrix 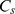 denote the neuronal response covariance matrix for stimulus *s*. Let the *N*×1 vector 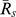 be the average population responses to stimulus *s* across 100 trials. Here we calculate the optimal weight vector 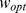 that yields maximum discrimination between stimuli. The *N* elements of the vector represent the weights applied to the response of individual neurons in the population. The optimal solution for the weight vector is obtained by maximizing the signal-to-noise ratio:
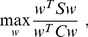
(1)where 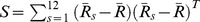 represents the *N×N* signal covariance matrix, *^T^* denotes the transpose operator, the *N*×1 vector 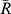 represents the average population responses across all stimuli (n = 12), and 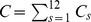 represents the overall neuronal trial-by-trial covariability. In Equation 1, the numerator is proportional to the population signal strength along the vector 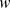, while the denominator is proportional to the noise along the vector 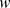. The signal to noise ratio calculated in this way is invariant under scaling 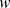. Thus we can always find an optimal weight vector 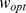 such that 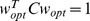. The maximization problem in Equation 1 is a quasi-convex optimization problem [11] with the following Lagrangian function:
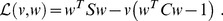
(2)Applying the Karush–Kuhn–Tucker conditions [11] yields:
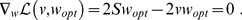
(3)where 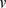 represents the Lagrange multiplier corresponding to the equality constraint. Assuming 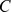 is invertible, Equation 3 can be restated as
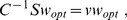
(4)which is equivalent to eigenvalue decomposition of 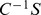 , where the optimal weight vector 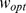 is along the eigenvector corresponding to the largest eigenvalue of 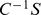 [8], [11].

An upper bound on the performance of the linear discrimination can be achieved by finding the optimum set of weights for every pairwise stimulus discrimination. In this condition, for the particular stimulus pair 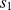 and 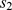 with average neuronal population responses 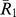 and 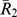 , and covariance matrices 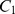 and 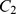, the signal-to-noise ratio along weight vector 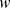 can be simplified to the following formula:
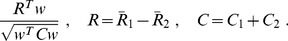
(5)Solving for the optimal weight which maximizes the above equation by applying the same approach as in the problem formulated in Equation 1 yields 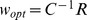 [8]. This solution is identical to linear least square error estimation of the two classes [8], [9]. The overall discriminability (*A*) for the whole stimulus set was defined as the mean value of *A* across all possible stimulus pairs (n = 66). Throughout the paper, we refer to this upper bound as the pairwise-optimal decoder.


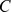
 is not invertible when at least one of the recorded neurons does not fire any spikes in response to any stimuli. Calculation of the optimal weight vector is generalized to conditions when 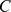 is singular, simply by removing the neurons with zero average spike count and then setting their corresponding weight to zero.

### Quantifying the decoder tolerance to deviation from the optimal weight

According to Equation 4, the solution for the optimal weight vector is the generalized eigenvalue decomposition of the signal covariance matrix 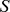, and the noise covariance matrix 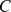. The problem can be transformed into a subspace where 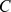 is invertible, and hence the optimal weight vector is the first eigenvector of 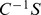. However, as 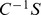 is not a symmetric matrix, other eigenvectors are not orthonormal. To quantify the level of tolerance of the decoder to changes in the weight vector direction, we need a symmetric representation of the effect of rotation in the space of neuronal activity with respect to the optimal direction. Thus we transpose the eigenvectors of 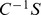 to an orthogonal basis by rotating the eigenvectors according to the Gram–Schmidt procedure.

### Quantification of signal and noise correlations

To characterize signal correlation in a population of more than two neurons, we applied principal component analysis (PCA) [12] on the z-scored neuronal average spike counts, similar to the quantification of noise correlation employed in [5]. For a population of *N* neurons, let the *N×12* matrix 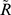 denote the z-scored average neuronal responses to stimulus set averaged across 100 trials. Below we show that signal correlation can be represented by the largest normalized eigenvalue of the neuronal response correlation matrix 
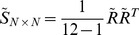
. The strength of the signal correlation is proportional to the amount of stretch in the joint distribution of the average population responses. The first eigenvalue of the signal correlation matrix – denoted by 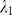 – normalized to the sum of all eigenvalues specifies the maximum covariation in the average z-scored population responses relative to all dimensions forming the space of population activity. Thus normalized 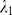 represents the stretch or skewness in the joint distribution of population responses, and hence identifies the signal correlation.

As the sum of all eigenvalues equals the sum of all diagonal elements of the signal correlation matrix 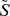, which is equal to *N*, the normalized 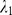 can be re-expressed as:
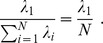
However, normalized 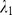 has a positive constant bias which depends on the number of neurons in the population and the number of stimuli: When population size, *N*, is less than 12 (the number of stimuli), the maximum number of non-zero eigenvalues of signal correlation matrix is *N*, and hence the minimum value of normalized 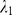 is 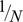. When population size is 12 or more the rank of signal correlation matrix is limited to 11. Thus the maximum number of non-zero eigenvalues of signal correlation matrix is 11, and hence the minimum value of normalized 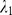 is 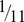. In order to provide a measure of signal correlation which is independent of population size or number of stimuli, we subtracted this bias from normalized 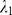 and rescaled the result such that it falls between 0 and 1. We define this measure as the signal correlation index, denoted by SCI:
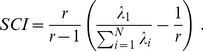
(6)where 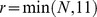. The signal correlation index depends solely on the correlation between the average responses of neurons. Similarly, the noise correlation index, denoted by NCI, is defined as [5]:
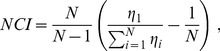
(7)where 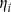 is the i^th^ greatest eigenvalue of the average noise correlation matrix across stimuli. For the special case of two neurons, signal and noise correlation indices are identical to the absolute value of the correlation coefficient between neuronal responses averaged across stimuli, and the correlation coefficient of trial-by-trial response variability, respectively [5].

## Results

The information that can be inferred from neuronal populations depends on the ‘readout mechanism’. A biologically plausible method of decoding applies a weight to each input neuron before integrating their response (Figure 1A). The weight coefficient represents the synaptic strength between the input neuron and the downstream decoder. A simple readout mechanism, called pooling, sums the activity of input neurons together with equal weights [13] (Figure 1B, left panel). At the other extreme, a decoder may only ‘read’ the activity of the most informative neuron in the population. This scheme, called the ‘lower envelope principle’ [14], [15], gives a weight of 1 to the best input neuron and a weight of zero to all other input neurons. Figure 1C compares the performance of these two decoding schemes applied to the neuronal responses to vibrotactile stimuli of different amplitudes (0 to 33 µm with equal increments of 3 µm), using the discriminability index, *A*. This index was averaged across all possible stimulus pairs (n = 66) in the non-adapted state. For some populations, pooling outperformed the best neuron, while in other populations pooling performance was not as good as the best neuron.

A third linear decoding scheme takes signal and noise correlations across neurons into account and finds the weights that optimize discriminability (Figure 1B, right panel) by maximizing the average signal-to-noise ratio (SNR). We will refer to this optimal linear decoder as the optimal decoder. Figure 1D quantifies pairwise stimulus discriminability across all possible populations of 8 simultaneously recorded single neurons in our dataset. The optimal decoder achieved a 96.8% improvement in discrimination performance over pooling, as quantified by the average value of *A*. In this decoding scheme, neurons with a higher SNR are expected to obtain a higher weight and thus make a greater contribution to decoding. To verify this, Figure 1E gives the distribution of weights as a function of SNR. As predicted, the decoder assigns weights of higher absolute value to the neurons with higher SNR.

**Figure 2:**
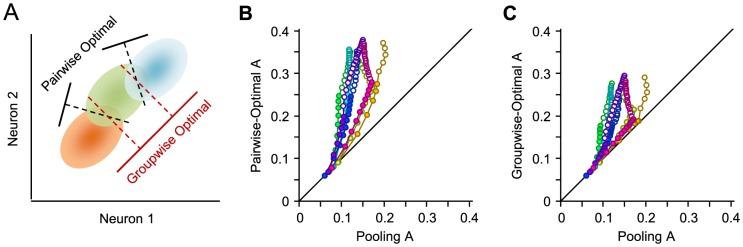
Pairwise- and groupwise-optimal decoding schemes. **A.** Schematic representation of pairwise- and groupwise-optimal decoding. **B.** The average value of *A* for a range of population sizes. Data from neuronal populations within a session are plotted with the same color and connected with a line. Filled markers correspond to populations of single units. For each population size of single units, the value of *A* of all possible selections of that size was averaged within session. Open markers correspond to further additions of multi-units to the whole population of single units. Thus the direction of increasing population size is from filled markers to open markers. For every population of mixed single and multi-units, the value of *A* was obtained by averaging across a maximum of 400 possible selections within a session. **C.** as in **B**, but for groupwise-optimal decoding.

Figure 2 generalizes the analysis to populations of various sizes. In Figure 2B a distinct set of weights were found for every stimulus pair, thus we refer to this decoder as the ‘pairwise-optimal decoder’. Pairwise-optimal decoding outperformed pooling with the effect becoming more pronounced at larger population sizes. In order to apply the appropriate set of weights, such a decoder requires *a priori* knowledge about the pair of stimuli to be discriminated. An arguably more biologically plausible decoding scheme is to apply an identical weight vector to discriminate across all stimulus pairs. By analogy with the pairwise-optimal decoder, we refer to this coding scheme as the ‘groupwise-optimal decoder’. Figure 2A provides a comparison of the two schemes. Figure 2C illustrates that the groupwise-optimal decoder outperformed pooling for every population size. Similar to the pairwise-optimal decoder, the improvement over pooling increases with population size. Across all population sizes, the groupwise-optimal decoder was superior to pooling by 54.8%±26.8% (mean ± s.d. across sessions). The rest of the analyses will focus on the groupwise-optimal decoding scheme.

### Robustness of the decoder

How well does the decoder generalize to new trials? To address this question, we obtained the optimal weight vector from half of the trials (100 random selections of 50 out of 100 trials), and then applied the weights to the other half. In this analysis, we first focus on populations of 8 simultaneously recorded single neurons as a sample population size. On average, the discriminability on untrained trials was 95.7%±1.7% (mean ± s.d. across sessions) that on trained trials. This level of generalization was not specific to the population size of 8 single neurons. Across sessions, the performance of the decoder on untrained trials was 96.8%±2.5% and 96.7%±1.7% of that on trained trials for the whole set of single neurons ranging from 6 to 11 across sessions, and for the whole set of single- and multi-units in each session, respectively.

We further quantified the extent to which the decoder approaches the maximum achievable discriminability in terms of the value of *A*. In order to do this, we numerically calculated the weight vector which directly maximizes the value of *A*, using the pattern-search optimization method [16]. We compared the performance of this decoder, *A*-optimum, with that of the groupwise-optimal decoder. The weight vectors for both approaches were obtained from half of the trials (100 random selections of 50 out of 100 trials), and then were applied to the remaining half. Across sessions, the performance of the groupwise-optimal decoder was 99.1%±3.2% of *A*-optimum (for the whole set of single neurons) and 99.5%±1.3% (for the combined set of simultaneously recorded single- and multi-units).

**Figure 3:**
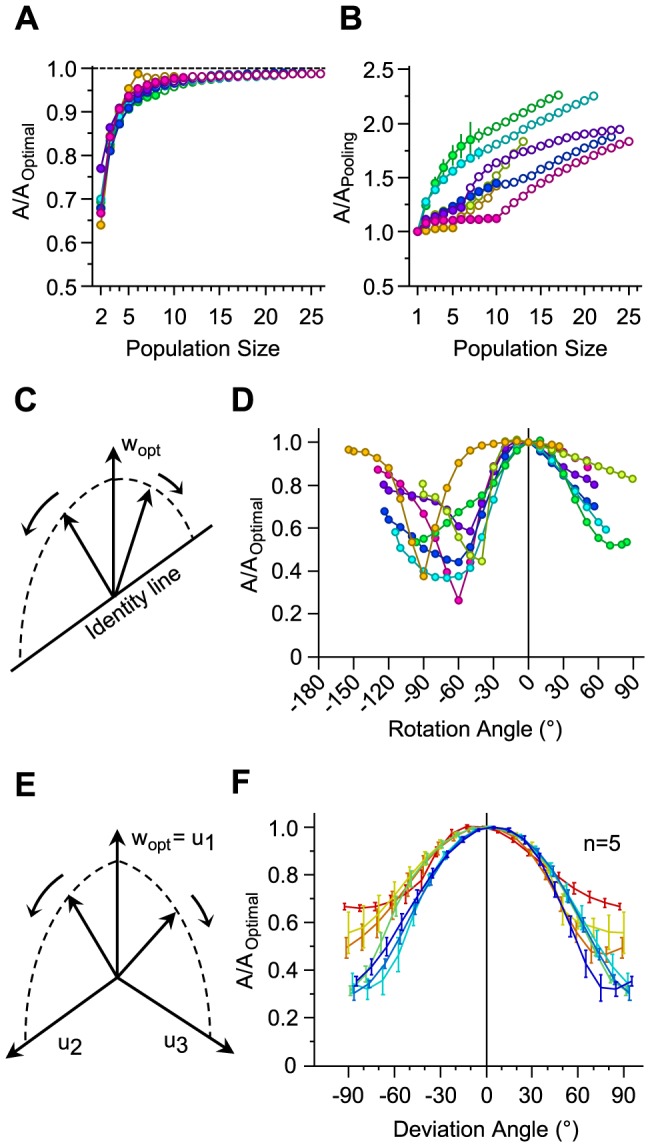
Decoder tolerance to weight vector deviation. **A.** The relative decline in the performance of the decoder after dropping a unit by setting its weight to zero. For every population, the performances were calculated and averaged across all possible 1-unit reductions. Plotting conventions are the same as in Figure 2. **B.** The performance of the decoder after dropping a unit relative to the performance of the pooling of the reduced population. The abscissa represents the size of the population after 1-unit reduction. **C.** Rotating the weights from the optimal direction towards the identity line which corresponds to pooling. Rotation was performed along two asymmetric paths. **D.** The relative change in the performance of the decoder when the weight vector was deviated from the optimal direction in steps of 10° towards the pooling direction (identity line). Every session is represented with a color and corresponding data points are connected with a line. The two extreme data points on each curve correspond to the performance of pooling relative to the groupwise-optimal decoder. For every session, the population of all single neurons was considered. **E.** Systematically rotating the weights from the optimal direction, denoted by 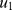, towards every other dimension in the space of neuronal activities. Dimensions are orthogonal, and span the space of the neuronal activity. The trajectory of this rotation lies on the 2-dimensional plane spanned by the optimal direction and the target dimension, and hence is perpendicular to all other dimensions. **F.** The relative performance of decoder with a deviated weight vector compared to the optimal direction for all possible populations of 8 neurons averaged across sessions (n = 5). Colors indicate the 7 trajectories toward associated dimensions, with red corresponding to the dimension perpendicular to optimal direction such that it maximizes the separation between neuronal responses, and blue corresponding to the dimension along which the separation between neuronal responses is minimal. Error bars indicate standard error of the means.

To what extent does the decoder tolerate a change in the weight vector? We first examine the relative contribution of individual weights by setting the weight of one unit to zero while maintaining the weight of the other units in the population. This is equivalent to removing one unit from the population. Figure 3A depicts the relative decline in the performance of this decoder (the suboptimal decoder) as a function of the original population size. At the population size of 2, the relative decline in the performance of the suboptimal decoder was 31.0%±1.5% (mean ± s.e.m. across sessions), it reached 5% for the population size of 7 single neurons, and diminished as the population size further increased. The suboptimal decoder still outperformed pooling for the reduced population (Figure 3B). The difference between the performance of suboptimal and pooling increased with population size. We further compared the performance of the suboptimal decoder with the decoder optimized on the reduced set of units. On average, across sessions and population sizes, the performance of the suboptimal decoder was 99.4% that of the optimal decoder, with a minimum of 98.6%±0.3% (mean ± s.e.m. across sessions) observed at the reduced population size of 2.

In order to further quantify the extent to which the decoder tolerates a change in the weight vector, we gradually rotated the weight vector from the optimal direction towards the identity line (Figure 3C). Since decoding along the identity line corresponds to pooling, this analysis provides a characterization of the transition from optimal to pooling. Figure 3D illustrates the effect of rotating the weight vector from optimal direction towards the identity line. As the optimal direction is not perpendicular to the identity line, the 180° trajectory of rotation is not symmetric, but longer on one side (see Figure 3C). The consequence is a minimum in performance at an angle close to 90° (maximum deviation from optimal). For each curve, the two ends of the trajectory correspond to pooling, which in general is neither the best nor the worst decoding strategy.

Setting the identity line as the endpoint of rotation provides an intuitive link between optimal decoding and pooling. However, this represents a specific and rather arbitrary trajectory of rotation. To further characterize the tolerance of the decoder, we systematically rotated the weight vector away from the optimal towards all *N-1* other dimensions in the *N*-dimensional space of population activity (Figure 3E). The optimal weight vector is the eigenvector of 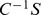 corresponding to the highest eigenvalue – where the separation between the population responses to the stimuli is maximal in the SNR space. Likewise, other dimensions correspond to the orthogonalized eigenvectors of 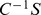 (see Methods). The separation of the population responses to the stimuli is correspondingly higher along an eigenvector with a higher eigenvalue. Accordingly, we expect the decoding performance to drop less when the weight vector is rotated toward an eigenvector with a higher eigenvalue. Figure 3F characterizes decoding performance when the weight vector is rotated towards each of the 7 dimensions corresponding to other eigenvectors for a population size of 8 single neurons. Performance dropped by 34.0%±2.9% (mean ± s.d. across sessions) along the second most informative dimension and by 69.7%±6.9% along the least informative dimension. Across all dimensions, for a 30° deviation, we observed an average drop of 10.2%±1.8% (mean ± s.d.).

### Effect of noise correlation on decoding

**Figure 4:**
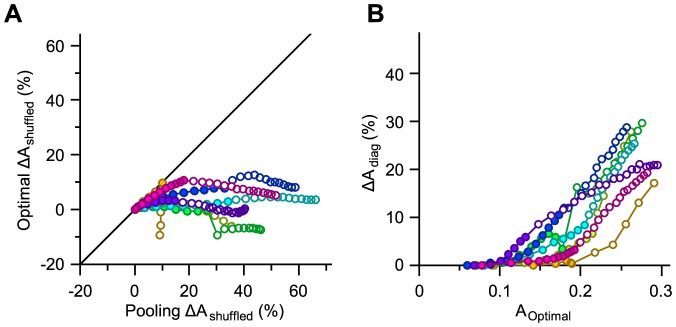
Effect of noise correlation on decoding. **A.** The ordinate represents the relative effect of noise correlation. This is captured by the per cent change in the value of *A* for a groupwise decoder optimized on trial-shuffled neuronal responses. The abscissa indicates the effect of noise correlation on pooling scheme. This is captured by the increase in the value of *A* for pooling after trial-shuffling neuronal responses. Color conventions and selection of neurons for every population are identical to Figure 2. For every selection of neurons for a given population size, the values of *A* for 50 trial-shuffles were averaged. **B.** The effect of ignoring noise correlation when decoding. *ΔA*_diag_ corresponds to the per cent drop in the performance of a decoder which ignores noise correlation. Noise correlations were ignored by setting the off-diagonal elements of the total covariance matrix to zero.

How does the trial-to-trial correlation in neuronal activity (i.e. the noise correlation) affect the performance of the decoder? To address this question, we first decorrelated the neuronal responses by shuffling the order of trials for every neuron in the population. Shuffling the trial orders eliminates neuronal response covariations while preserving the marginal distribution of population responses and the signal correlation. Thus, any observed effect of trial-shuffling is entirely due to noise correlations. We quantified the effect of noise correlation by *ΔA*_shuffled_ denoting the percentage difference between the performance of the decoder optimized on the trial-shuffled responses and the performance of the decoder optimized on the true neuronal responses. Previous analysis [5] revealed that neuronal covariability is positive and thus detrimental to the information content of the pooled neuronal responses. Therefore, removing noise correlation is expected to enhance decoding performance. For pooling, removing noise correlation systematically improved decoding, as expected (Figure 4A). This effect increased with pool size, reaching an average improvement of 44.0% in the value of *A* across sessions in our dataset. However, for optimal decoding, removing noise correlation had no systematic effect, sometimes improving and sometimes impairing performance (the average change in the value of *A* across sessions was 0.9%, ranging from −9.6% to 12.5% for any population size in our dataset).

The immunity of the optimal decoder to the presence of noise correlation implies that the decoder has incorporated the structure of neuronal covariability. To directly test this idea, we implemented a simpler decoding scheme in which the covariance matrix was forced to be diagonal such that only signal correlation and the variability of individual neurons contributed to the optimization. This is equivalent to optimizing the decoder on the decorrelated population responses and then applying the resulting weights on the true population responses. We denote the value of *A* for this decoding scheme by *A*_diag_. Figure 4B plots the proportional drop in the average value of *A* as a result of ignoring noise correlation, as denoted by *ΔA*_diag_. *ΔA*_diag_ increased with population size, reaching 30% in our dataset. This finding reveals that the decoder successfully accounts for the noise correlation.

### Adaptive population decoding

How does sensory adaptation affect the information content of neuronal populations? The original data set contained not only the non-adapted responses analyzed thus far, but also responses collected under two states of adaptation (vibration amplitudes of 6 and 12 µm). This allowed us to investigate how well the optimal linear readout performs under adaptation compared to the non-adapted state. The functional specialty of the whisker-barrel system and the structure of somatosensory cortex as a stand-alone processing stage in rodents [17] suggest that cortical neurons may have access to the network dynamics and the adaptation state. This information can be exploited to optimize the readout under different states of adaptation, leading to an ‘adaptive decoding scheme’.

**Figure 5:**
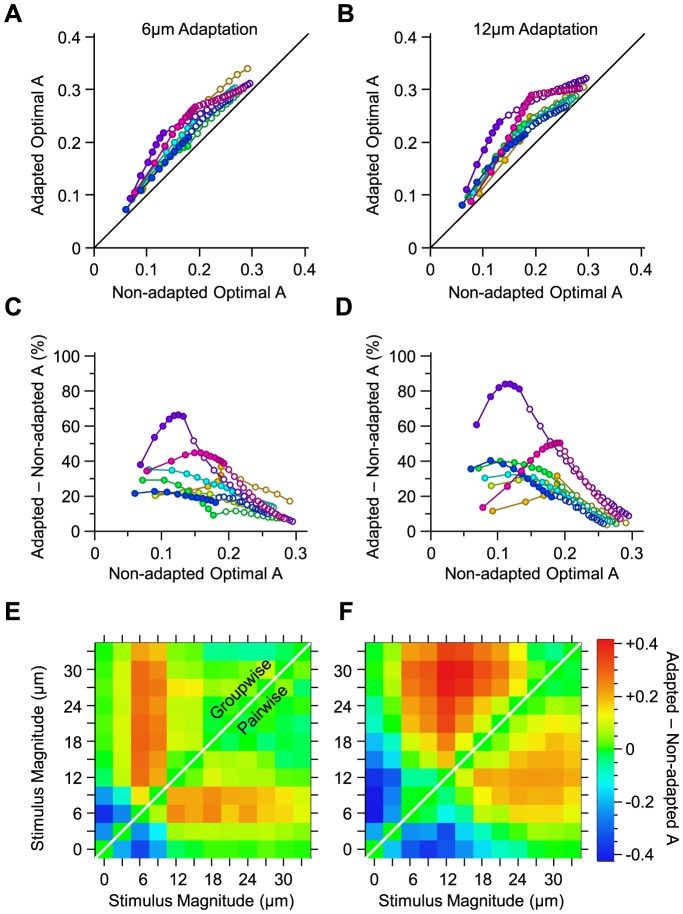
Adaptive optimal decoding. **A.** The value of *A* for the optimal decoder when optimized on population responses under 6 µm adaptation, against the value of *A* for the optimal decoder when optimized on non-adapted population responses. **B.** As in **A**, but for 12 µm adaptation. **C.** The per cent improvement in the value of *A* under 6 µm adaptation relative to the non-adapted state. **D.** As in **C**, but for 12 µm adaptation. **E.** The effect of 6 µm adaptation on the value of *A* for every stimulus pair, in a sample session with 11 simultaneously recorded single neurons, indicated by purple color in **A**–**D**. The upper triangle corresponds to the groupwise-optimal decoder, while the lower triangle corresponds to the pairwise-optimal decoder. **F.** As in **E**, but for 12 µm adaptation.

Figure 5A–D quantify the discrimination performance of an adaptive optimal decoder (in terms of the average value of *A*) under different adaptation states. The average value of *A* for optimal decoder is higher under adaptation compared to the non-adapted state. This improvement is most prominent at intermediate *A* values and diminishes at low and high levels of discrimination performance (Figure 5A and B). These results extend the finding that sensory adaptation enhances coding efficiency from pooling [5] to optimal linear integration.

The enhanced discriminability demonstrated in Figure 5C and D is the average improvement across all pairwise stimulus discriminations (n = 66). To elucidate how sensory adaptation affects the coding efficiency for different stimuli, we quantified the adaptation-induced change in the value of A for individual stimulus pairs. As illustrated in Figure 5E and 5F, there is an elevated discriminability for stimuli higher in amplitude than the adaptor, while there is a decline in discriminability for stimuli lower than the adaptor. This pattern was consistent across sessions (correlations for all pairwise comparisons across sessions were significant, with all p values<0.008, and an average correlation coefficient of 0.68), as well as across both groupwise and pairwise optimal decoding schemes (correlation coefficient between average values across sessions: 0.97). The magnitude of the effect was larger for the groupwise optimal decoder compared with the pairwise decoder (linear regression coefficient of 1.14, significantly higher than 1 with a p value<0.05, regression R^2^>0.93). Additionally, the peak magnitudes of the decline and the enhancement were close: respectively, −0.23±0.05 (mean ± s.e.m. across sessions) vs. 0.20±0.04 for 6 µm adaptation, and −0.28±0.04 vs. 0.26±0.04 for 12 µm adaptation. These findings represent a shift in discriminability from low amplitudes to amplitudes higher than the adaptor [18], and are consistent with the lateral shift in the amplitude response function of the population [5]. For both adaptation states, the number of stimulus pairs for which discriminability increased was higher than the number of stimulus pairs for which it declined. This led to a net increase in the average value of A.

In order to understand the nature of this improvement in coding efficiency, we quantify the modulation of sensory adaptation on the two components of our optimization objective function (SNR): signal and noise correlations. We then parse out the contribution of each component (signal and noise) to the improvement in coding efficiency through adaptation.

### Effect of noise correlation on adaptive decoding

**Figure 6:**
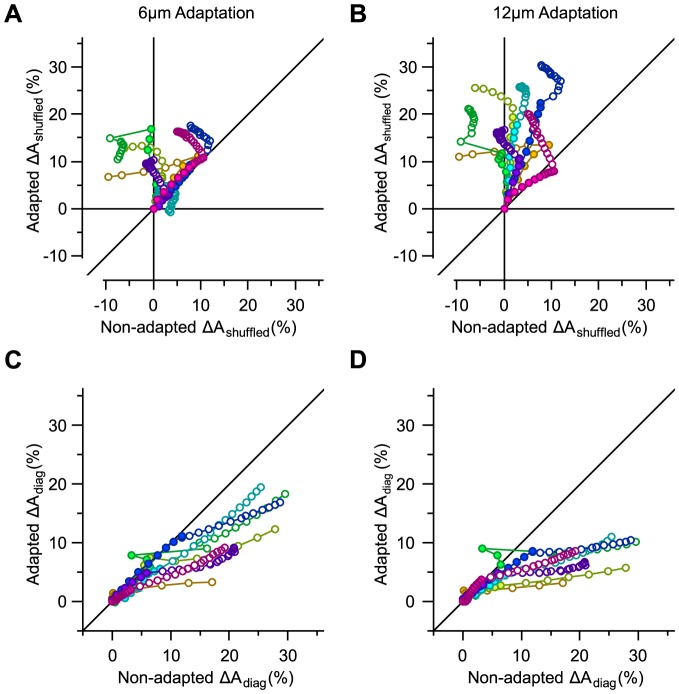
Effect of noise correlation on the adaptive optimal decoder. **A.** The per cent change in the value of *A* by trial-shuffling, denoted by *ΔA*_shuffled_, for 6 µm adaptation, against the same measure in the non-adapted state. **B.** As in **A**, but for 12 µm adaptation. **C.** The per cent drop in the value of *A* by ignoring noise correlation when decoding, denoted by *ΔA*_diag_, for 6 µm adaptation, against the same measure in the non-adapted state. **D.** As in **C**, but for 12 µm adaptation.

What is the functional effect of noise correlation on the performance of the optimal decoder under different adaptation states? To address this question, Figure 6A and B illustrate *ΔA*_shuffled_ for the two adapted states and compare it with the non-adapted state. The comparison reveals two main findings. First, the magnitude of the effect of noise correlation was greater in the adapted state. For instance, on average, across populations of 8 single neurons, noise correlation degraded decoding by 3.8%±3.7% (mean ± s.d. across sessions) in the non-adapted state, 8.9%±4.7% in the 6 µm adaptation state and 14.6%±5.1% in the 12 µm adaptation state. This finding is consistent with the results in our previous study that adaptation increased the overall noise correlation [5]. The second difference is in the functional role of noise correlation: contrary to the observation in the non-adapted state whereby noise correlation exhibited positive as well as negative effects on decoding efficiency (abscissa in Figure 6A and B), noise correlation was always detrimental to decoding under adaptation (ordinate in Figure 6A and B). This was in spite of the fact that the decoder was optimized on the adaptation data.

Based on this result, one might expect that ignoring noise correlation to be more detrimental to the performance of a decoder in the adapted state. However, this was not the case. Figure 6C and D illustrate the proportional drop in the value of *A* when ignoring noise correlation – as captured by *ΔA*_diag_. The detrimental effect of ignoring noise correlation on decoding was less under adaptation. We explore two hypotheses to explain this discrepancy. Sensory adaptation might modulate the population responses in two ways: (i) increase in signal correlation and (ii) decrease in the angle between signal and noise direction. The following section quantifies signal and noise correlations for populations of any size.

### Effect of adaptation on signal correlation

What is the effect of sensory adaptation on the redundancy of neurons? As a measure of response redundancy, we quantified the correlation in the average responses to the stimuli, or signal correlation, under each adaptation state. A widely-used measure of signal correlation in the literature is the correlation coefficient between the response functions of two neurons [1], [2], [19]–[26]. However, the cross correlation analysis could not be applied to dimensions beyond two neurons. Therefore, we further scrutinized the correlations in the average response of multiple neurons with principal component analysis (PCA). In mathematical terms, the first eigenvector of the average neuronal spike-count correlation matrix identifies the direction of the greatest correlated variability (signal direction), and the first eigenvalue, denoted by 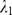, signifies the magnitude of that variability. The value of 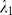 normalized to all eigenvalues quantifies the degree of the stretch in the population responses and thus the strength of signal correlation.

**Figure 7:**
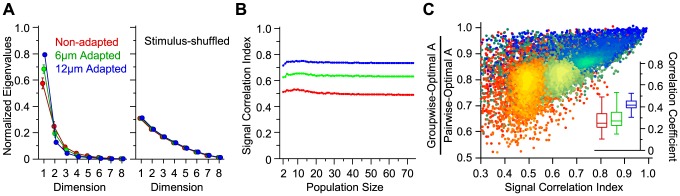
Effect of adaptation on signal correlation. **A.** The eigenvalues of the signal correlation matrix for populations of 8 simultaneously recorded neurons, sorted into descending order. Each eigenvalue was normalized to the sum of all eigenvalues. Error bars indicate standard error of the means across sessions (n = 5). The colors red, green and blue correspond to the non-adapted state, 6 µm adaptation and 12 µm adaptation, respectively. The right panel illustrates the normalized eigenvalues for stimulus-shuffled neuronal responses. The stimulus labels for each neuron in a given population were randomly shuffled 100 times, and the corresponding eigenvalues were averaged. **B.** Signal correlation as captured by the signal correlation index as a function of population size under the three adaptation states. For population sizes 72 and 73, all possible selections of neurons (n = 73 and 1, respectively) and for the other population sizes 500 random selections of neurons were used to obtain the signal correlation index. Color convention is identical to **B**. **C.** The performance ratio of groupwise-optimal decoder to pairwise optimal decoder as a function of signal correlation index for the populations in **B**. Different population sizes are plotted in different levels of brightness with the colors red, green and blue corresponding to the non-adapted state, 6 µm adaptation and 12 µm adaptation, respectively. Each data point corresponds to one population. The inset shows the distribution of correlation coefficients calculated per population size for each adaptation state.

We first focus on sample populations of 8 simultaneously recorded single units. Figure 7A shows the 8 eigenvalues of the signal correlation matrix for the stimulus set across the five sessions that contained 8 single units or more. Normalized 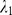 captures over 57.4% of the covariations in the average population responses to the stimuli in the non-adapted state. The first three eigenvalues represent 91.4% of stimulus-driven cross-neuronal response variability. This is a consequence of the similarity in the intrinsic response pattern of cortical neurons to stimulus intensity; a sigmoidal increase with stimulus intensity [7], [27]. Normalized 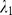 was higher in the adaptation states compared to the non-adapted state. This finding supports the prediction that sensory adaptation increases signal correlation. This increase in signal correlation is achieved principally by alignment of neuronal response functions through a lateral shift in the amplitude response function of individual neurons [5]. Shuffling the labels of stimuli across neurons reduced signal correlation and essentially eliminated the difference between adaptation states (right panel in Figure 7A). This confirms that the adaptation-induced increase in normalized 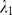 is not confounded by the sampling structure of neuronal responses, or the response variability of individual neurons in the population, but is a direct consequence of signal correlations across neurons in the population.

As signal correlation analysis captures correlations in the ‘average’ response of neurons across trials, it can also be applied to neurons that were not recorded simultaneously. Thus, we applied this analysis across all single-units (n = 73) in our dataset. Figure 7B represents signal correlation across various population sizes up to 73 single neurons. For this analysis, we calculated the signal correlation index – a rescaled version of normalized 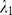 adjusted for population size (see Methods). The signal correlation index exhibits a constant relationship with population size signifying that this index is not biased by the number of neurons in the population. The value of signal correlation index was higher in the adapted states, revealing that sensory adaptation increased the homogeneity of cortical neuronal response functions.

We used the same method to quantify noise correlations for the simultaneously recorded units, as explained in detail in [5]. On average, adaptation increased the signal correlation index more than noise correlation index by factors of 4.6 and 3.0 (medians across sessions) for 6 µm and 12 µm adaptation states, respectively. This explains the observed improvement in the performance of the decoder with sensory adaptation. In addition, this result reveals why ignoring noise correlation is less detrimental under adaptation (see Figure 6). We also quantified the angle between the signal and noise direction under each adaptation state and observed no systematic changes across adaptation states. Likewise, across sessions, there was no systematic change in the first eigenvector of signal covariance matrix, 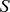 with respect to the first eigenvector of the net noise covariance matrix, 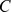, over the three states of adaptation.

The performance of the groupwise-optimal decoder approaches its upper bound, pairwise-optimal decoder, when the neuronal responses to sensory stimuli are linearly correlated. This is equivalent to a maximal signal correlation. In this situation, provided that the noise direction is essentially invariant with stimulus, the direction of the optimal weight vector for every stimulus pair is identical, and lies along the groupwise-optimal weight vector. This indicates that the signal correlation can be captured as the difference in the performance of the pairwise and groupwise optimal decoding schemes. Figure 7C verifies this relationship by quantifying the correlation between the signal correlation index and the ratio of the groupwise- to pairwise-optimal decoding performance (Pearson correction coefficient of 0.94; *p*<0.0001). For over 99% of population sizes and adaptation cases, the correlation coefficient between signal correlation index and the ratio of the groupwise to pairwise optimal decoders' value of *A* was significant (*p* values<0.05).

**Figure 8:**
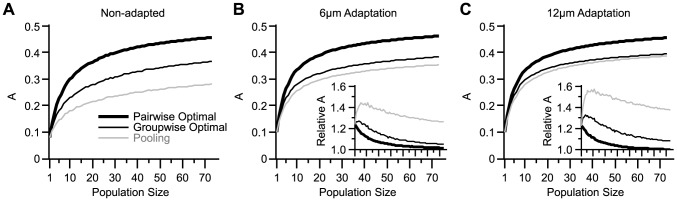
Performance of various coding schemes under different states of adaptation. **A.** The discrimination performance of the pairwise-optimal decoder (solid thick black curve), the groupwise-optimal decoder (solid thin black curve) and pooling (gray line) in the non-adapted state, as a function of population size. For every population size the value of *A* was averaged across 500 random selections of single neurons from all recorded single neurons (n = 73). For population sizes 1, 72 and 73 the possible distinct selections from 73 single neurons were 73, 73 and 1, respectively. Thus for these population sizes the value of *A* was averaged across all possible selections. **B.** As in **A**, but for the 6 µm adaptation. The inset represents the relative change in the average value of *A* through sensory adaptation. **C.** As in **B**, but for 12 µm adaptation.

The increased signal correlation through sensory adaptation leads to the following prediction: as a result of the increased homogeneity in neuronal response curves, under adaptation pooling is expected to be closer to the optimal decoding. We tested this prediction in the absence of noise correlation. Figure 8 summarizes different decoding schemes as a function of population size. For each population size, the neurons were selected randomly from all recording sessions. For those neurons in the population that were recorded simultaneously, if any, we shuffled the order of trials in order to eliminate the noise correlation. As predicted, under adaptation, pooling was closer to groupwise-optimal decoding. Adaptation enhanced the performance of all decoding schemes; however this improvement declined with population size (see insets in Figure 8). The improved decoding efficiency was most prominent for pooling.

### Non-adaptive decoding vs. adaptive decoding

**Figure 9:**
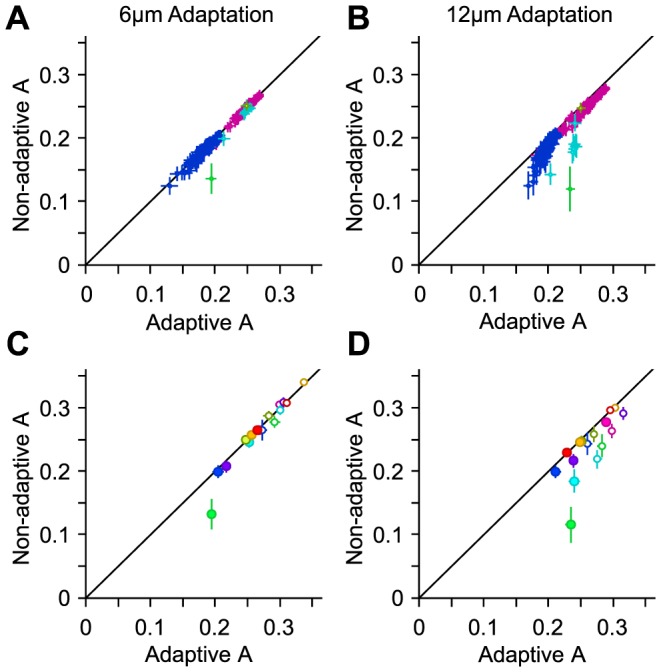
Decoding generalization across adaptation states. **A.** The abscissa indicates the value of *A* for the optimal decoder when optimized on half of the adapted responses and tested on the other half for 6 µm adaptation across populations of 8 single neurons. The ordinate corresponds to the value of *A* for the optimal decoder when optimized on half of the non-adapted responses and tested on the same half of the adapted responses as in the abscissa. Error bars indicate standard error of the means. Colors indicate different sessions. **B.** As in **A**, but for 12 µm adaptation. **C.** As in **A**, but for whole session populations. Filled markers represent populations comprising single units only, while open markers indicate the whole set of simultaneously recorded single- and multi-units in a session. **D.** As in **C**, but for 12 µm adaptation.

To what extent does the non-adaptive decoder generalize across states of adaptation? Figure 9 addresses this question by using a fixed set of weights optimized in the non-adapted state. This figure compares the decoding performance of the non-adaptive decoder with the adaptive optimal decoder under each adaptation state. For this comparison, the performances were always quantified using untrained trials. To quantify the performance of the adaptive decoder, non-overlapping sets of training and test trials were obtained from the same adaptation state. For the non-adaptive decoder, the training trials were selected from the non-adapted state while the test trials were from the adapted state. For the 6 µm adaptation state, non-adaptive performance was 93.2%±13.0% (mean ± s.d. across sessions) that of the adaptive decoder for all possible populations of 8 single neurons (Figure 9A). For the 12 µm adaptation state, non-adaptive performance was 83.5%±19.8% that of the adaptive decoder (Figure 9B). In addition to populations of 8 single neurons, we further investigated the level of decoder generalization across adaptation states for the whole set of simultaneously recorded single neurons, as well as the whole set of single- and multi-units in each session. On average, across sessions, for the 6 µm adaptation state, non-adaptive performance was 94.7%±10.9% and 99.3%±2.5% that of the adaptive decoder, for each population set respectively (Figure 9C). For the 12 µm adaptation state, non-adaptive performance was 88.0%±17.4% and 91.5%±7.1% that of the adaptive decoder (Figure 9D).

**Figure 10:**
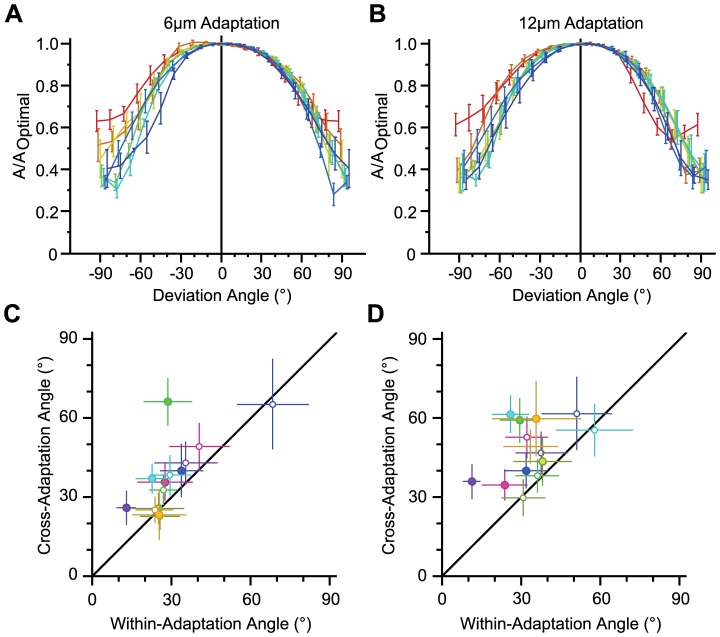
Adaptive and non-adaptive decoding tolerance to weight vector deviation. **A.** The relative performance of adaptive decoding with a deviated weight vector compared to the optimal direction for all possible populations of 8 neurons averaged across sessions (n = 5) for 6 µm adaptation state. Colors indicate the 7 trajectories toward associated dimensions, with red corresponding to the dimension perpendicular to optimal direction such that it maximizes the separation between neuronal responses, and blue corresponding to the dimension along which the separation between neuronal responses is minimal. Error bars indicate standard error of the means. **B.** As in **A**, but for 12 µm adaptation. **C.** Abscissa represents the angular difference of optimal weight vectors for non-overlapping trial-halves within the 6 µm adaptation state. Ordinate represents the angular difference for 6 µm adaptation versus non-adapted state. For each session, the angular differences were averaged across 100 times of random trial-halving. Error bars indicate standard error of the means. Colors indicate different sessions. **D.** As in **C**, but for 12 µm adaptation.

This level of cross-adaptation generalization could either indicate that the performance of the adaptive decoder is relatively insensitive to changes in weights, or that adaptation does not strongly affect the optimal weights. To investigate this, we first quantified the sensitivity of the adaptive decoder to deviation of the weight vector from its optimal value in the adapted conditions. We systematically rotated the weight vector away from the optimal direction towards all *N-1* other dimensions in the *N*-dimensional space of population activity (see Figure 3E). Figure 10A and B demonstrate the sensitivity of the adaptive decoder for a population size of 8 single neurons. For both adaptation conditions, the discriminability of the decoder consistently degraded with the angle of deviation. Consistent with the non-adapted condition (Figure 3F), the drop in the value of A was greater along the less informative dimensions compared with the more informative dimensions; performance dropped by 36.9%±11.1% and 46.0%±9.8% (mean ± s.d. across sessions) along the second most informative dimension and maximally dropped by 71.8%±11.8% and 64.9%±10.5% towards the least informative dimension for the 6 µm adaptation and 12 µm adaptation, respectively. Across all dimensions, for a 30° deviation, we observed an average drop of 4.7%±1.5% (mean ± s.d.) for the 6 µm adaptation, and a drop of 5.6%±1.3% for the 12 µm adaptation, which is less than the 10.2%±1.8% drop for a 30° deviation in the non-adapted case.

We further quantified the angular difference between the non-adaptive decoding weight vectors and the adaptive one for 6 µm and 12 µm adaptation states. The angular difference directly quantifies the effect of adaptation on the signal and noise directions, and its subsequent effect on the optimal weight vector. Figure 10C and D demonstrate the angular difference between the adaptive decoding weight vector and the non-adaptive one, in terms of the inverse cosine of their dot product. This measure is always positive, leading to a potential positive bias in the estimation of the average angular difference. To estimate this bias, we measured the angular difference between optimal weight vectors of the non-overlapping trial-halves within the adapted state. We then analyzed the correlation between the level of generalization (as in Figure 9) and the bias-subtracted angular differences across states of adaptation. The correlation analysis revealed an anti-correlation between the two measures (Pearson correlation coefficient: −0.6029, p = 0.0007); the higher the generalization across states of adaptation, the lower the changes in the weight vectors.

## Discussion

Here, we characterized the performance of a readout mechanism that linearly combines the responses of neurons in rat barrel cortex. The coefficients of this linear combination represent the synaptic weights between the barrel cortex neurons and the downstream neuron (decoder). We found the weights that maximized the average signal-to-noise ratio taking into consideration correlated variability across neurons. Such a decoder was less sensitive to noise correlations and adaptation state compared to a simple pooling method. In contrast to pooling, where noise correlation was always detrimental to the information content of the pooled population responses, for some populations noise correlation improved the optimal decoding performance. This motif is consistent with similar results of a recent study which quantified texture discrimination accuracy of cortical population responses in awake rats just prior to behavioral responses [28]. Under the optimal coding scheme, the response of less informative neurons could be exploited to provide information about the network state and the structure of noise correlations. We found that adaptation increased noise correlation [5], leading to a greater effect of noise correlation on decoding than in the non-adapted state (Figure 6A and B). Ignoring noise correlation led to a decline in the decoder performance (Figure 4B). This decline increased with population size. Although noise correlation was stronger under adaptation, ignoring it during decoding was less detrimental to the decoding efficiency. This was mainly due to a greater increase in signal correlation through sensory adaptation.

In the present study, we characterized the pairwise discrimination performance using a criterion-free metric, *A*, in the framework of signal detection theory. Fisher information between neuronal responses and stimuli provides an alternative measure of discriminability [29]–[33]. Fisher information averaged across stimuli is proportional to the value of *A* when averaged across stimulus pairs with minimum difference (3 µm in our study) – see [33], [34]. Furthermore, we found that the optimal decoding scheme that maximized the signal-to-noise ratio (and not directly the value of *A*), did identify the maximal value of *A* (see Figure 3F).

We employed two parallel methods in order to quantify the effect of noise correlation on the information content of the population responses; (1) the effect of trial shuffling on discriminability index, as captured by *ΔA*_shuffled_, directly quantifies the effect of noise correlation on coding efficiency, and (2) *ΔA*_diag_ quantifies the cost of ignoring noise correlation. These measures are analogous to information theoretic measures such as *ΔI*_shuffled_ [5], [35]–[37] and I_cor-dep_ [38]–[43], as well as other measures based on signal detection theory such as *Δd^2^*_shuffled_ and *Δd^2^*_diag_ [44]–[46].

Along the lemniscal pathway, there is a greater than 10 fold increase in the number of neurons representing a whisker from brainstem to cortex; from 160–200 neurons per barrelette [47] and 250–300 neurons per barreloid [48]–[50] to about 2500 cortical neurons per layer IV barrel [51], [52]. One explanation for this increase might be the need to represent multiple features (e.g. a broad range of speeds of whisker motion). For example, the broad range of perceptually discriminable whisker motions [7], [53] can be broken down into narrower ranges. Each of these narrowed ranges of whisker motion intensities could then be represented by a subpopulation of neurons sensitive to that range. The weights of neurons for these combinations could be optimized using the solution applied in the present study. Further experiments are required to investigate the mechanism through which such optimal synaptic weights could potentially be developed across multiple subpopulations.

An important question is whether the readout mechanism adjusts to changes in neuronal response dynamics. This question is not limited to sensory adaptation. In addition to adaptation (temporal context), spatial context can also modulate the response properties of neurons [54], [55] and produce similar perceptual biases and illusions [32]. Likewise, attention also changes the tuning properties of neurons [56]–[59] and induces perceptual illusions [60], [61]. The match between perceptual predictions based on a non-adaptive decoder and psychophysical measures of perceptual biases and thresholds in the visual system is consistent with a fixed non-adaptive readout [32], [33]. However, several attributes of an adaptive readout could potentially produce similar perceptual biases [33]. In addition, cortical neurons may be able to provide information about network dynamics and adaptation state to downstream structures. Further experiments are required to quantify the psychophysical effect of sensory adaptation in the whisker-mediated touch system in rodents.

Here, we observed a remarkable cross-adaptation generalization. In isolation, this could either indicate that the decoding performance is relatively insensitive to changes in weights or that adaptation does not strongly affect the optimal weights. Given the dependence of the decoding performance on the changes in weights as revealed in Figure 10A and B, we conclude that the optimal weights remain relatively unchanged after adaptation. These results can be understood in terms of the changes in the response function of cortical neurons through sensory adaptation. Sensory adaptation shifted the response function and response variability profile of cortical neurons with no systematic modulation on the response saturation level [5]. Thus the set of weights, which maximize discriminability between a pair of stimulus amplitudes in the non-adapted state, are expected to maximize discriminability between a new pair of stimulus amplitudes that are in effect simply shifted by the adaptor.

Our previous study showed that sensory adaptation increases noise correlation across neurons [5]. This increase in noise correlation tends to decrease the overall signal-to-noise ratio. The marked level of cross-adaptation generalization indicates that signal correlation across neurons increases with sensory adaptation as well. This increase in signal correlation can be explained in terms of the adaptation-induced lateral shift in the response of single neurons. In the non-adapted state, neurons exhibit various sensitivity thresholds. However, sensory adaptation tends to equalize the threshold of neurons by aligning their response functions with respect to the adapting stimulus amplitude [5]. This response alignment homogenizes the population of neurons, leading to increased signal correlation.

Here, decoding was performed along the first eigenvector of the 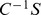. The decoding scheme can however be expanded to other eigenvectors of 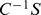. As these eigenvectors are not orthonormal (see Methods), the information along them is correlated, leading to redundant population coding. An interesting question is how sensory adaptation changes the direction of these eigenvectors and the amount of information along them in a multi-dimensional feature space of sensory stimuli. If through sensory adaptation the eigenvectors rotate away from each other to form a more orthogonal basis, the information extracted from them is less correlated, leading to an adaptive decorrelated representation of sensory features along these eigenvectors [62], [63].

In the present study, and also in previous relevant studies [28], [36], [46], [64]–[72] the decoder is commonly optimized to maximize the discriminability or minimize the estimation error. However, a behaviorally-relevant question is “which readout mechanism matches the perceptual accuracy of subjects?” To address this question, the optimization objective function should be set to a behavioral measure such as choice probability [73]. Investigating such a perceptually-matched decoder under different temporal (adaptation), spatial or attentional contexts would reveal the extent to which the readout adjusts to context-induced changes in neuronal response dynamics.
